# OP35 Early Access To Medicinal Products In France: A Positive Evaluation Two Years Into The New Framework

**DOI:** 10.1017/S0266462324000977

**Published:** 2025-01-07

**Authors:** Virginie Crespel, Guillaume Roux, Alice Desbiolles, Alexandre Beaufils, Thierno Diatta, Floriane Pelon, Camille Thomassin

## Abstract

**Introduction:**

Introduced in July 2021, early access to pharmaceuticals allows French patients to have rapid and reimbursed access to presumed innovative medicines prior to marketing authorization or regular reimbursement registration. The French National Authority for Health (Haute Autorité de Santé [HAS]) is responsible for granting early access authorizations and for health technology assessment upstream of regular reimbursement. This analysis evaluates the outcomes of the 2021 reform.

**Methods:**

In collaboration with the national regulatory body (ANSM) and the Ministry of Health, a descriptive and retrospective analysis has been conducted to assess the impact of this reform. The data were extracted from published HAS decisions and from the HAS internal information system (EVAMED). The period considered is the first two years of the reform, from 1 July 2021 to 30 June 2023. Different information has been analyzed, such as conclusions of decisions returned, therapeutic areas concerned, and decision processing time. An additional analysis focused on HAS opinions on regular reimbursement for the medicines for which early access was granted.

**Results:**

In two years, over 250 applications (including first applications, modifications, and renewals) have been submitted, increasing over time, and 180 decisions were issued by HAS within this period. Eighty percent of HAS decisions were positive for early access. Among the medicines for which early access was granted, 86 were also assessed for regular reimbursement, of which 78 percent obtained a positive clinical added value score (at least ASMR IV). Overall, the early access program is estimated to have facilitated coverage approximately nine months before regular reimbursement and benefited over 100,000 patients.

**Conclusions:**

Manufacturers make extensive use of early access applications. A positive assessment of the reform outcomes emerges from this analysis, emphasizing the role of HAS in accelerating patient access to presumed innovative treatments. In light of US experience and of the uncertainties surrounding fast-track authorization procedures, monitoring of the French early access system will continue.

